# Distinct Characteristics of Odor-evoked Calcium and Electrophysiological Signals in Mitral/Tufted Cells in the Mouse Olfactory Bulb

**DOI:** 10.1007/s12264-021-00680-1

**Published:** 2021-04-15

**Authors:** Han Xu, Chi Geng, Xinzhong Hua, Penglai Liu, Jinshan Xu, Anan Li

**Affiliations:** 1 Jiangsu Key Laboratory of Brain Disease and Bioinformation, Research Center for Biochemistry and Molecular Biology, Xuzhou Medical University, Xuzhou, 221004 China; 2 School of Medical Information and Engineering, Xuzhou Medical University, Xuzhou, 221004 China

**Keywords:** Mitral/tufted cells, Fiber photometry, Electrophysiology, Odor representation, Olfactory bulb

## Abstract

Fiber photometry is a recently-developed method that indirectly measures neural activity by monitoring Ca^2+^ signals in genetically-identified neuronal populations. Although fiber photometry is widely used in neuroscience research, the relationship between the recorded Ca^2+^ signals and direct electrophysiological measurements of neural activity remains elusive. Here, we simultaneously recorded odor-evoked Ca^2+^ and electrophysiological signals [single-unit spikes and local field potentials (LFPs)] from mitral/tufted cells in the olfactory bulb of awake, head-fixed mice. Odors evoked responses in all types of signal but the response characteristics (e.g., type of response and time course) differed. The Ca^2+^ signal was correlated most closely with power in the β-band of the LFP. The Ca^2+^ signal performed slightly better at odor classification than high-γ oscillations, worse than single-unit spikes, and similarly to β oscillations. These results provide new information to help researchers select an appropriate method for monitoring neural activity under specific conditions.

## Introduction

Electrophysiological signals recorded *in vivo* reflect neuronal activity directly with high temporal resolution. Extracellularly-recorded activity ranges from single-unit spikes to neuronal populations [e.g., local field potentials (LFPs)] and has been used extensively in both anesthetized and awake behaving animals [1, 2]. For many years, it has been considered the gold standard for measuring neural activity [2, 3]. Fiber photometry has the ability to record neuronal activity in genetically-defined cell types in specific brain areas, including both cortex and sub-cortical nuclei [4], but monitors the population Ca^2+^ signal; these recordings thus indirectly reflect the activity of a specific population of neurons. Fiber photometry has been widely used to monitor the activity of cell-type-specific populations and to correlate activity with specific behaviors [5–8]. Fiber photometry has several advantages over extracellular electrophysiology, including low noise, low cost, and relatively simple implementation, and has been used extensively in neuroscience studies of sensory processes, motor behavior, learning, memory, and cognition [5].

Olfaction plays a crucial role in survival, helping animals find food and avoid predators, amongst other functions. Olfactory dysfunction has been found in the early stages of several nervous system disorders, including Alzheimer's disease, Parkinson's disease, and depression [9–12]. As the first processing center in the olfactory system, the olfactory bulb (OB) receives input from sensory neurons, processes this information, and then transmits it to higher centers, such as the anterior olfactory nucleus, olfactory tubercle, and piriform cortex [13–16]. The OB also receives feedback from the olfactory cortex and centrifugal innervation from cholinergic, noradrenergic, and serotonergic cells [14, 17, 18]. In the OB, the mitral/tufted cells (M/Ts) are the main output neurons; M/T activity is regulated by interneurons, including granular cells and juxtaglomerular cells [14, 19]. Functionally, M/Ts are critically involved in the representation of odor identity, value, intensity, and timing [20, 21]. Given the complicated networks in the OB and the importance of the M/Ts, efficient methods to sensitively and specifically monitor M/T neural activity are crucial for olfactory research.

In the OB, both spikes and LFP signals recorded from the M/Ts are widely used to investigate its role in the representation of odor information and olfactory learning and memory [22–24]. However, with spike/LFP recording there is no direct genetic evidence to prove that the recorded spikes are from M/Ts rather than other cell types, although indirect evidence can be provided by the firing properties [25–27]. Fiber photometry has been used in the OB and piriform cortex to investigate neural responses to odor stimulation under different brain states [6, 28–30]. In the OB, robust odor-evoked responses are recorded in M/Ts and granule cells [6, 28]. Thus, fiber photometry is emerging as an efficient and popular method to study cell function in the OB. However, because it monitors neural activity indirectly, it is critical to correlate the Ca^2+^ signals recorded *via* fiber photometry with the gold-standard measurement of neural activity.

In this study, we used a mouse model with M/T-specific GCaMP6s expression to simultaneously record odor-evoked Ca^2+^ signals and electrophysiological signals from M/Ts in awake, head-fixed mice. We examined the response characteristics, correlations, and odor-decoding ability of the different types of signal. Our findings show how Ca^2+^ signals recorded by fiber photometry relate to electrophysiological recordings and have implications for the application of fiber photometry throughout the brain.

## Materials and Methods

### Animals

Eleven male Thy1-cre [FVB/N-Tg(Thy1-cre)1V1n/J] mice aged 8–16 weeks were used in this study. The mice were bred in the animal facilities of Xuzhou Medical University and housed in a vivarium under a 12 h/12 h light/dark cycle, with lights on at 08:00. Experiments were performed during the light cycle. After surgery, the mice were housed individually for at least 10 days before further experiments to allow recovery. Food and water were available *ad libitum*. All experimental procedures were performed in accordance with protocols submitted to and approved by the Xuzhou Medical University Institutional Animal Care and Use Committee.

### Fabrication of Opto-tetrodes

The opto-tetrodes included one optical fiber and four tetrodes. The optical fiber (200 μm O.D., NA = 0.37, 18.5 mm long) was coupled with a ferrule (10.5 mm long; 2.5 mm in diameter). Each tetrode consisted of four polyamide-coated nichrome wires (single-wire diameter, 12.7 μm; coating 1/4 hard PAC; Sandvik, RO–800, item # PF000591) gold-plated to an impedance of 0.2–0.3 MΩ. The tetrodes and optical fiber together were inserted into tubing (450 μm O.D., 320 μm I.D., 9.0 mm long, A-M Systems), which was glued to an EIB-16 interface board (Neuralynx). The ends of the tetrodes were connected to the interface board with gold pins (large size, Neuralynx); the shafts were glued to the side of the optical fiber and then the tetrodes were cut so that the tetrode tips and the optical fiber tip were at the same level.

### Virus Injection

The virus injection procedure was similar to that described in our previous studies [6, 28]. Briefly, in Thy1-cre mice, the mitral cell layer of the OB was injected with AAV-DIO-GCaMP6s virus (PT-0071, AAV2/9, 5.03×e+12 vg/mL, BrainVTA, Wuhan, China). All injections were made with a glass pipette and the injection volume and velocity were controlled by a microsyringe pump (Quintessential Injector; Stoelting Co.). Virus solutions (300 nL) were injected at 30 nL/min into the mitral cell layer (4.2 mm anterior, 1.0 mm lateral, and 0.8–1.1 mm ventral to bregma). The glass pipette was left in place for an additional 10 min before being slowly withdrawn. After the viral injection, the scalp was sutured. Mice were individually housed for at least three weeks after surgery for recovery and to allow time for the expression of GCaMP6s.

### Surgery for Implantation of Opto-tetrodes

Mice were briefly anesthetized with pentobarbital (0.09 mg/g bodyweight, i.p.). Depth of anesthesia was verified by toe pinch. Next, each mouse was mounted in a stereotaxic frame and the fur on the surface of the scalp from the midline of the orbits to the midpoint between the ears was removed. A hole was drilled above the right OB for the implantation of opto-tetrodes (4.2 mm anterior from bregma, 1.0 mm lateral from the midline). One screw hole was drilled into the parietal bone to serve as the ground and reference electrode in spike and LFP recordings.

Opto-tetrodes were implanted into the OB with the aim of simultaneously recording Ca^2+^ signals, single-unit spikes, and LFPs. The opto-tetrodes were lowered to the lateral mitral cell layer at an average depth of 1.8–2.0 mm. Recordings were made during opto-tetrode implantation to ensure optimal placement within the lateral mitral cell layer. The signals recorded from the tetrodes were sent to a headstage, amplified by a 16-channel amplifier (Plexon DigiAmp; bandpass filtered at 1–5000 Hz; 2000× gain), and then sampled at 40 kHz by a Plexon OmniPlex recording system. In order to secure mice in the head-fixed recording system, an aluminum head plate was attached to the skull with stainless steel screws and dental cement.

### Odor Presentation

Eight odorants (isoamyl acetate, 2–heptanone, phenyl acetate, benzaldehyde, dimethylbutyric acid, n–heptane acid, n–pentanol, 2–pentanone; from Sinopharm Chemical Reagent Co. and Sigma-Aldrich, USA) were presented by an odor delivery system (Thinkerbiotech, Nanjing, China). All odorants were dissolved in mineral oil at 1% v/v dilution. In the odor delivery period, a stream of nitrogen flowed over the oil at 100 mL/min, and was then diluted to 1/20 by an olfactometer. Odor presentation was synchronously controlled by the data acquisition system *via* a solenoid valve that was driven by the digital-to-analog converter. Air or odorized air was delivered to the nose at a constant rate of 1 L/min to eliminate the effect of airflow. For each odor, 20 trials were presented with an inter-trial interval of 30 s. The duration of each odor presentation was 2 s. Odors were presented passively: mice were head-fixed and awake but were not required to produce any behaviors in response to odor presentation and did not receive any reward.

### Histology

To verify viral expression, frozen brain sections were prepared. Mice were anesthetized with pentobarbital (80 mg/Kg bodyweight, i.p.) and transcardially perfused with 20 mL of 0.9% saline, followed by 20 mL of 4% paraformaldehyde (PFA) in PB (0.1 mol/L, pH 7.4). After perfusion, each brain was harvested, postfixed for 24 h in PFA at 4°C, and then cryoprotected with 30% sucrose in PBS until the tissue sank. The brain was then embedded in OCT compound and sectioned at 30 μm on an upright Leica cryostat. Tissue sections were mounted on slides and imaged by a confocal scanning microscope (Zeiss, LSM710).

### Calcium Signals, Spikes, and LFP Recordings

Before recording the Ca^2+^ signals, spikes, and LFPs, mice were required to have recovered and be in good condition for 10 days after the surgery. Two horizontal bars (fixed to the head plate by 2 screws) were used to head-fix awake mice, which were able to maneuver on an air-supported free-floating Styrofoam ball (Thinkerbiotech). After the mice had adapted for a period of time, Ca^2+^ signals, spikes, and LFPs were recorded simultaneously.

A fiber photometry system (ThinkerTech) was used to record fluorescence emissions. A laser beam from a laser tube (488 nm; OBIS 488LS; Coherent) was reflected by a dichroic mirror, focused through a 10× objective lens (NA = 0.3; Olympus) and then coupled to an optical commutator (Doric Lenses). An optical fiber (200 mm O.D., NA: 0.37, 1.5 m long) guided the light between the commutator and the implanted optical fiber. Laser power was modulated to 40–60 μW at the tip of the optical fiber. GCaMP6s fluorescence emission was band-pass filtered (MF525-39, Thorlabs) and detected by a photomultiplier tube (R3896, Hamamatsu). An amplifier (C7319, Hamamatsu) was used to convert the photomultiplier tube current output to voltage, which was further filtered through a low-pass filter (35 Hz cutoff; Brownlee, 440). The analog voltage signals were digitized at 500 Hz and recorded by fiber photometry software.

*In vivo* electrophysiological data from the tetrodes were sent to the headstage and amplified by a 16-channel amplifier (Plexon DigiAmp; bandpass filtered at 0.1–5000 Hz; 2000× gain) and sampled at 40 kHz by a Plexon OmniPlex recording system. The procedure for spike recordings was similar to that for recordings made during the tetrode implantation described above. The LFP signals were amplified (2000× gain; Plexon DigiAmp), filtered at 0.1–300 Hz, and sampled at 1 kHz. Odor stimulation event markers were recorded alongside the spike/LFP data *via* the Plexon OmniPlex recording system.

### Data Analysis and Statistics

#### Off-line Spike Sorting and Statistics of the Unit Data

Spikes were sorted and identified in Offline Sorter V4 software (Plexon). The separation of different units was performed by principal component analysis. A unit was classified as a single unit if <0.75% of the interspike intervals were <1 ms, as in previous studies [23, 31]. This resulted in unimodal firing rate distributions. The data 2 s before and 6 s after each odor stimulation event were extracted, and the mean firing rate (MFR) was generated by averaging the firing rate in 50-ms bins (Fig. 2B4). The spontaneous firing rate was calculated by averaging across the spikes fired during the 2 s before odor stimulation and the odor-evoked firing rate was calculated by averaging across the spikes fired during the 2 s after the onset of odor stimulation. To test for odor-evoked responses, we compared the area under the receiver operating characteristic curve (auROC) for the baseline firing rate with that for the odor-evoked firing rate across all trials for each cell–odor pair (Fig. 2C4). See below for details of the ROC and auROC calculations.

#### Analysis of LFP Signals

MatLab was used to analyze the LFP signals. Similar to previous studies, LFP signals were divided into four frequency bands: theta (2–12 Hz), beta (15–35 Hz), low gamma (36–65 Hz) and high gamma (66–95 Hz) [23, 28]. Since odors usually evoke strong and reliable changes in the power in the beta and high-gamma bands in awake animals [23, 24, 28], we focused on these bands. To assess the odor-evoked beta and high-gamma responses, we analyzed the signals from 4 s before to 6 s after the onset of odor stimulation. The wavelet transform method with the Morlet wavelet was used to compute the signal power spectral density over time (MatLab function ‘cwt’). For each trial, the baseline was normalized to 1; normalized trials were averaged for each odor (Fig. 2B2, B3). As with the spike statistics, we used auROC to estimate whether the odor evoked a significant response (Fig. 2C2, C3).

#### Analysis of Fiber Photometry Data

Data were exported as MatLab .mat files for further analysis. The data were segmented at the onset of odor stimulation within individual trials. We derived the values of fluorescence change (ΔF/F) by calculating (F−F_0_)/F_0_, where F_0_ is the baseline fluorescence signal averaged over a 2-s baseline time window before odor stimulation (Fig. 2A1, lower). ΔF/F values are presented as heatmaps or average plots. We also used auROC to estimate whether the odor evoked a significant response (Fig. 2B1).

#### ROC Analysis

ROC analysis was used to assess the responses evoked by odors. ROCs were implemented in MatLab software. The auROC is a nonparametric measure of the discriminability of two distributions. We used auROC to assess the neural responses to 8 odors. The value of auROC was defined as ranging from 0 to 1. A value of 0.5 indicates completely overlapping distributions, whereas a value of 1 indicates perfect discriminability. auROC values <0.25 were defined as inhibitory responses; auROC values >0.75 were defined as excitatory responses; 0.25 < auROC values < 0.75 were defined as no significant response. The latency of the response onset, the latency of the peak response, and the response duration were calculated from the auROC values (Fig. 3A–C). Onset latency was defined as the time at which the odor-induced response began (auROC <0.25 or >0.75). Peak latency was defined as the time at which the odor-induced response reached its maximum or minimum point. Response duration was defined as the time from the start to the end of the odor-induced response.

AuROC was also used to calculate the difference between two odor-induced responses. Two responses were randomly selected from 8 odor-induced responses in the same animal. auROC values were positively correlated with the difference in odor-induced responses (Fig. 6A, B).

#### Logistic Regression Classifier

To assess the discriminability of odor-induced Ca^2+^ and electrophysiological signals, logistic regression classifiers imported from Scikit-learn v0.21.3 were used to measure odor classification accuracy. All odor-induced responses were processed by subtracting the baseline and binned into 50-ms bins over the 0–5s after the onset of odor stimulation. The feature vectors used for training and testing were concatenated sets of binned responses and have been standardized. To evaluate the performance of four types of signal on odor discrimination, two were randomly selected from 8 odor-induced neural responses, and the scores were obtained by the average classification accuracy of 28 odor pairs based on 10-fold cross-validation.

### Statistics

Data are expressed as mean ± standard error (mean ± SE). The Anderson–Darling test was used to assess the normality of the data. All experimental data were non-normal. The Wilcoxon signed-rank test was used to test for differences between two paired samples. When there were more than two paired samples, the Friedman test was used, and the Tukey method was used for subsequent comparisons. The Kruskal–Wallis test was used for more than two independent samples, and the Tukey method was used for subsequent comparisons. The two-sample Kolmogorov–Smirnov test was used to determine whether data distributions differed between two groups. *P* <0.05 indicated a statistically significant difference. For correlation analysis, we calculated the absolute value of Pearson's linear correlation coefficient:

$$ rho\left( {a,b} \right) = \frac{{\mathop \sum \nolimits_{i = 1}^{n} (X_{a,i} - \overline{X}_{a} )\left( {Y_{b,i} - \overline{Y}_{b} } \right)}}{{\left\{ {\mathop \sum \nolimits_{i = 1}^{n} \left( {X_{a,i} - \overline{X}_{a} } \right)^{2} \mathop \sum \nolimits_{j = 1}^{n} \left( {Y_{b,j} - \overline{Y}_{b} } \right)^{2} } \right\}^{1/2} }} $$

## Results

### Odor-evoked Response Characteristics of Calcium Signals and Electrophysiological Signals in M/Ts

**Fig. 1:**
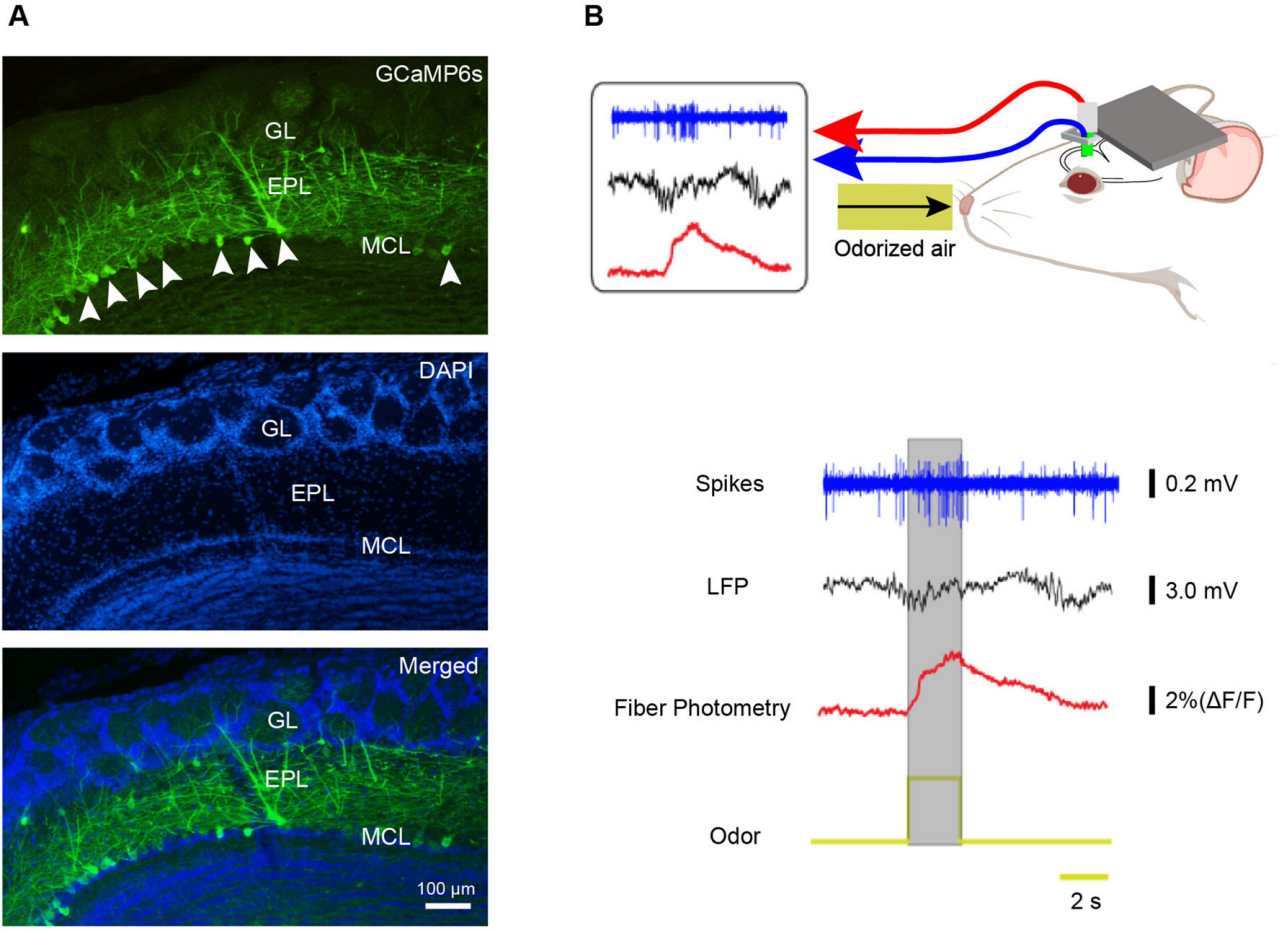
Simultaneous recording of odor-evoked spikes, LFPs, and population Ca^2+^ signals from M/Ts. **A** Expression of GCaMP6s (green) in M/Ts after injection of Cre-dependent GCaMP6s into the MCL of Thy1-Cre mice (arrowheads, mitral cells expressing GCaMP6s; GL, glomerular layer; EPL, external plexiform layer; MCL, mitral cell layer; scale bar, 100 µm). **B** Schematic of the recording set-up in awake head-fixed mice and example traces (gray box, 2-s odor-presentation period).

To specifically express the Ca^2+^ indicator GCaMP6s in M/Ts, we injected AAV-DIO-GCaMP6s-GFP into the mitral cell layer of the OB in Thy1-Cre mice. As in our previous studies [6, 32], GCaMP6s was specifically and efficiently expressed in M/Ts three weeks after the viral injection (Fig. 1A). To simultaneously record Ca^2+^ signals and electrophysiological signals, we implanted opto-tetrodes into the mitral cell layer of the OB two weeks after the viral injection. After waiting at least 10 days for recovery, we simultaneously recorded Ca^2+^ signals, single-unit spikes, and LFPs in response to a 2-s odor presentation in awake, head-fixed mice (Fig. 1B).

**Fig. 2:**
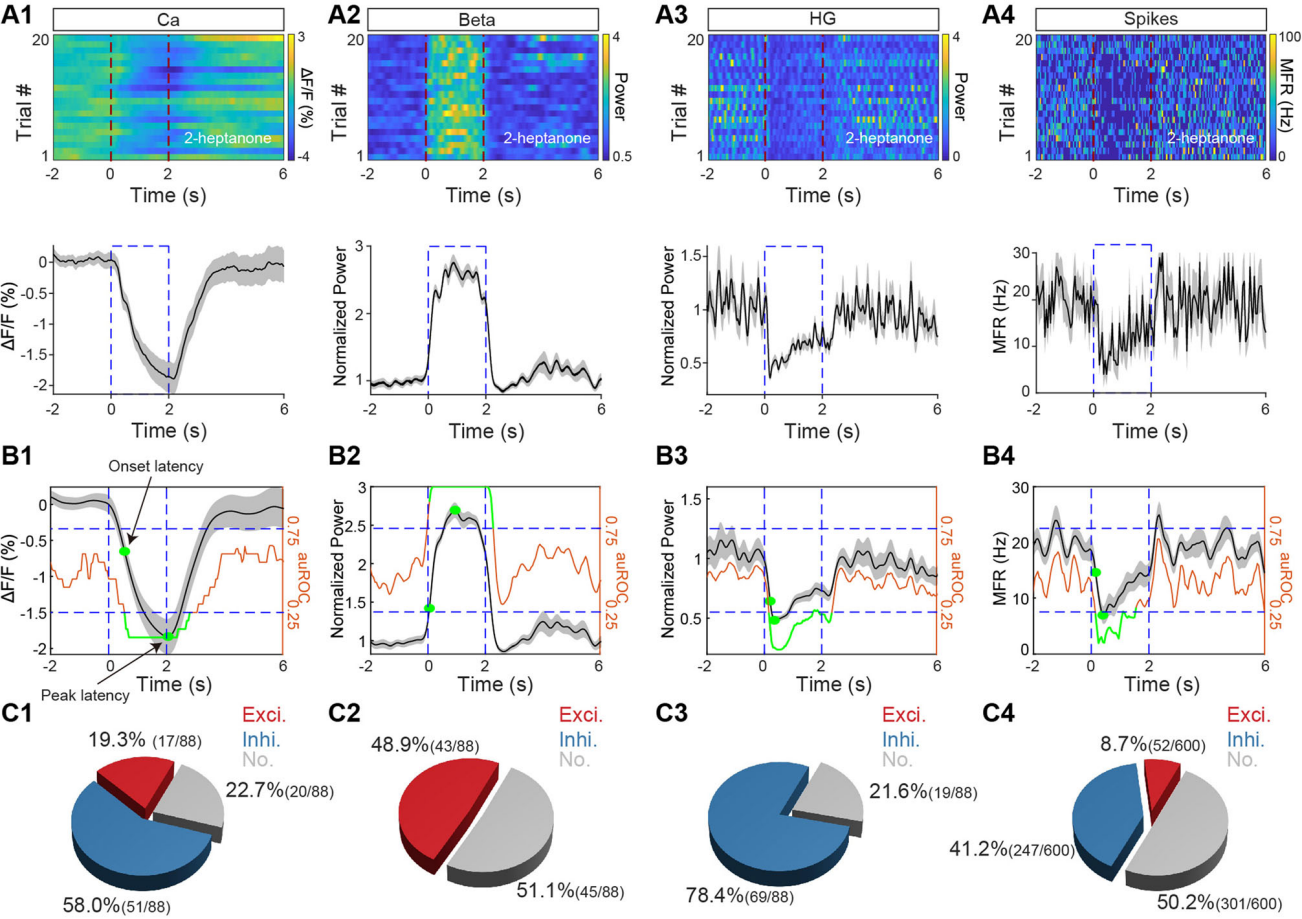
Odor-evoked Ca^2+^ signals, LFPs, and spikes in M/Ts**. A1**–**A4** Heat maps (upper panels, 20 trials, each row represents a single trial) and trial-averaged traces (lower panels) for the M/T fiber photometry Ca^2+^ signal, power in the beta and high-gamma LFP bands, and mean spike firing rate (MFR) evoked by one of 8 odors in a representative mouse (Ca, calcium; HG, high gamma). **B1**–**B4** auROC (brown lines) for ΔF/F, normalized power in the beta and gamma bands, and MFR (green lines, response duration; green dots, onset and peak latencies; black/gray lines, trial-averaged traces from one mouse/cell–odor pair). **C1**–**C4** Proportions of mouse/cell–odor pairs producing an excitatory (red), inhibitory (blue), or no (gray) response in the Ca^2+^ and electrophysiological signals (*n* = 88 mouse–odor pairs from 11 mice for Ca^2+^ signals and for beta and high-gamma oscillations, and *n* = 600 cell–odor pairs from 11 mice for spikes).

In the OB, odors usually evoke increased power in LFP beta oscillations and decreased power in high-gamma oscillations [6, 23]. These two types of oscillation are known to play different roles in odor information processing [24, 33]. Thus, we focused on the beta and high-gamma oscillations in the LFP. Heat maps of the Ca^2+^ signal, beta and high-gamma power, and spike firing rate during 20 odor-stimulation trials to one odor are shown in Fig. 2A (upper), with the averaged traces shown in Fig. 2A (lower). In this example, the odor evoked clear reductions in the Ca^2+^ signal, power in the high-gamma band, and the spike firing rate, but a robust increase in the power in the beta band. To quantitatively assess the odor-evoked responses in the four signal types, we used auROC to define whether the response increased, decreased, or did not change, and to determine the onset latency of the response, the latency to the peak response, and the response duration for significant odor-evoked responses (Fig. 2B; see Materials and Methods for details). For all the animals recorded (*n* = 88 mouse–odor pairs from 11 mice for Ca^2+^ signals and beta and high-gamma oscillations, and *n =* 600 cell–odor pairs from 11 mice for spikes), we found that odor evoked a significant response for all four types of signal (Fig. 2C). However, while both increased and decreased responses were recorded for the Ca^2+^ signals and spike rates (Fig. 2C1, C4), only increased responses were found for the beta oscillations (Fig. 2C2) and only decreased responses for the high-gamma oscillations (Fig. 2C3). These findings are consistent with results from previous studies in which these signals were recorded separately [6, 23].

**Fig. 3:**
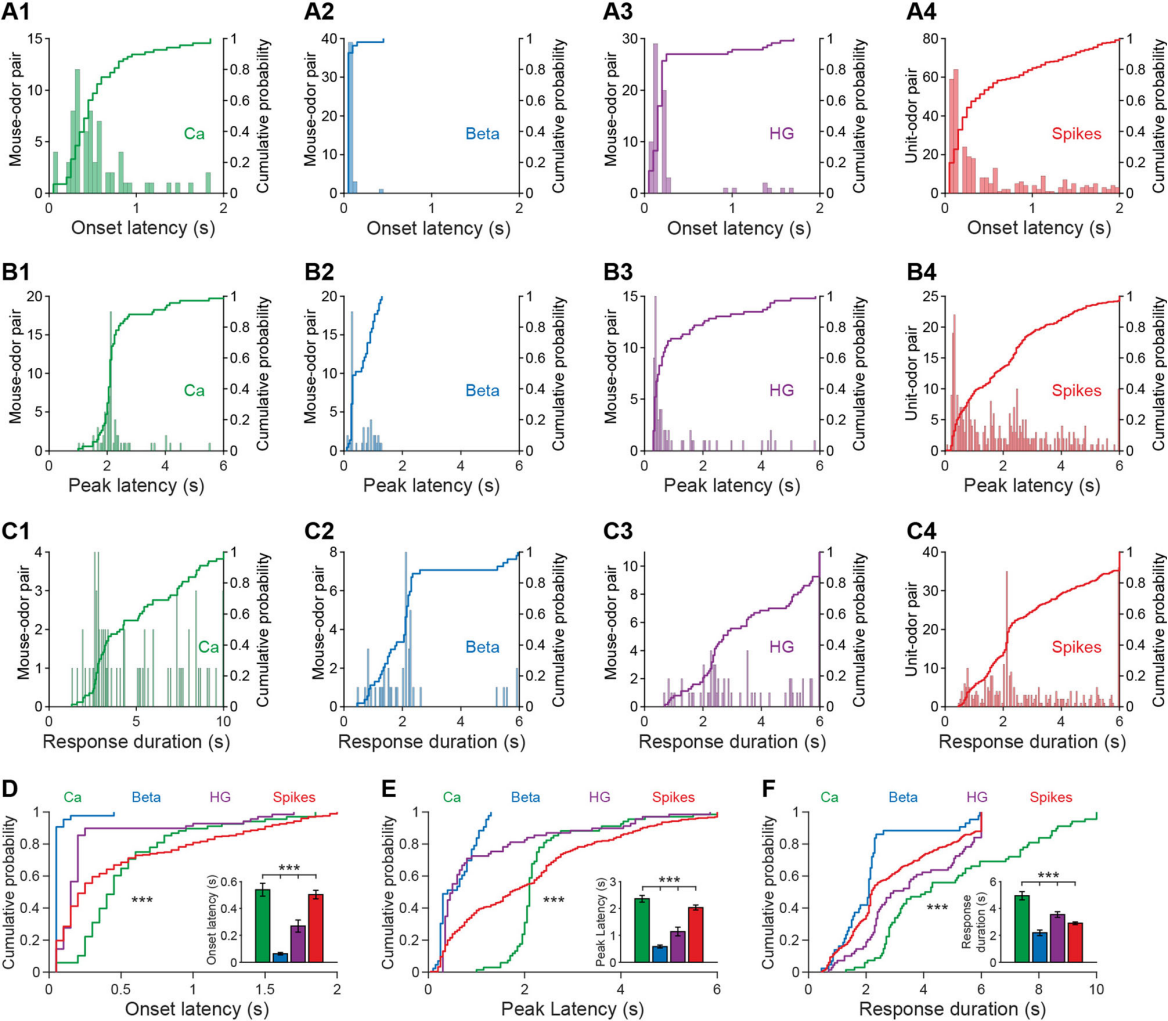
Odor-evoked temporal characteristics of the Ca^2+^ signals and electrophysiological signals. **A**–**C** Histograms and cumulative probability for onset latency (**A1**–**A4**), peak latency (**B1**–**B4**), and response duration (**C1**–**C4**) of the Ca^2+^ signal, beta oscillations, high-gamma oscillations, and spikes from all the mouse/cell–odor pairs (*n* = 68, 43, 69, and 299 for Ca^2+^, beta, high-gamma, and spikes, respectively). **D**–**F** Line charts of the cumulative probabilities for onset latency, peak latency, and response duration (two-sample K–S test, ***P <0.001). Bar charts (inset) show the mean values for onset latency, peak latency, and response duration (Kruskal-Wallis test, ***P <0.001). Ca, calcium; HG, high gamma.

Next, to further investigate the odor-evoked temporal characteristics of the four types of signal, we calculated the onset latency, peak latency, and response duration of the significant odor-evoked responses (both increases and decreases) (Fig. 2B) and compared them among the four types of signal (Fig. 3A–C). We found that the order for onset latency was Ca^2+^ signals > spikes > high-gamma oscillations > beta oscillations (Fig. 3D, Kruskal–Wallis test; Ca^2+^ signals *vs* spikes, *P* = 0.001; spikes *vs* high-gamma, *P* = 0.014; high-gamma *vs* beta, *P* = 0.014; *n* = 68, 43, and 69, from 11 mice for calcium signals, beta, and high-gamma, respectively, and *n =* 299 from 11 mice for spikes). The order for peak latency was Ca^2+^ signals > spikes > high-gamma/beta oscillations (Fig. 3E, Kruskal–Wallis test; Ca^2+^ signals *vs* spikes, *P* = 0.022; spikes *vs* high-gamma, *P* = 0.007; *n* = 68, 43, and 69 from 11 mice for Ca^2+^ signals, beta, and high-gamma oscillations, respectively, and *n* = 299 from 11 mice for spikes), with no significant difference in the peak latency for high-gamma and beta oscillations (Fig. 3E, Kruskal–Wallis test; *P* >0.05; high-gamma: *n* = 69 from 11 mice, beta: *n* = 43 from 11 mice). For response duration, the order was Ca^2+^ signals > high-gamma oscillations > spikes/beta oscillations (Fig. 3F, Kruskal–Wallis test, Ca^2+^ signals *vs* high-gamma, *P* = 0.027; high-gamma *vs* spikes, *P* = 0.012; *n* = 68, 43, and 69 from 11 mice for Ca^2+^ signals, beta and high-gamma oscillations, respectively, and *n* = 299 from 11 mice for spikes), with no statistical difference in the response duration for spikes and beta oscillations (Fig. 3F, Kruskal–Wallis test, *P* >0.05, spikes: *n* = 299 from 11 mice; beta: *n* = 43 from 11 mice). Thus, these findings indicate that the temporal characteristics of odor-evoked Ca^2+^ signals and electrophysiological signals are largely different, although some similar characteristics were found among the different types of electrophysiological signal.

### Correlation Between Calcium Signals and Electrophysiological Signals

**Fig. 4:**
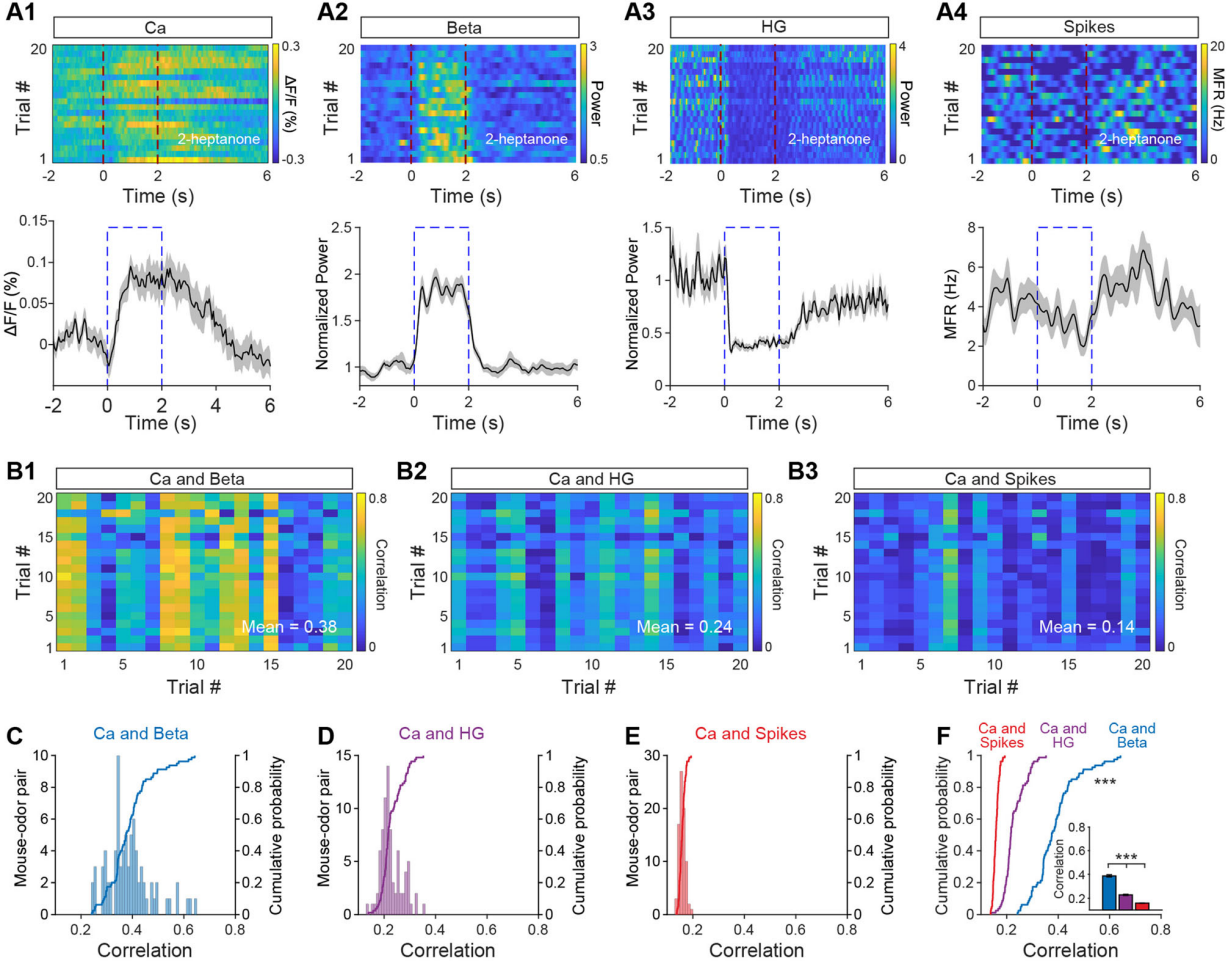
Correlations between the population Ca^2+^ signals and the electrophysiological signals. **A1**–**A4** Heat maps (upper panels, 20 trials, each row represents a single trial) and trial-averaged traces (lower panels) for the M/T Ca^2+^ signal, power in the beta and high-gamma LFP bands, and mean spike firing rate evoked by one of 8 odors in a representative mouse (dashed lines, odor-presentation period). **B1**–**B3** Correlations between the Ca^2+^ and the different electrophysiological signals. The matrices represent the correlation coefficients between the Ca^2+^ and electrophysiological signals evoked in individual trials by one of the odors (2-heptanone), in a representative mouse. **C**–**E** Histograms and cumulative probability of the correlation coefficients between the Ca^2+^ signals and the electrophysiological signals from all the mouse/cell–odor pairs (*n* = 80 from 11 mice). **F** Statistical analysis of the correlation coefficients shown in **C**, **D**, and **E** (line charts: two-sample K–S test, ***P <0.001; Bar chart: Kruskal-Wallis test, ***P <0.001). Ca, calcium; HG, high gamma.

Since the Ca^2+^ signals recorded by fiber photometry indirectly reflect neural activity from a population of specific neurons, it is important to assess how they correlate with the spikes and LFPs that directly measure neural activity, even though there are some differences in response types and time course between Ca^2+^ signals and electrophysiological signals. We analyzed the correlation between Ca^2+^ signals and spikes/beta/high-gamma oscillations using the Pearson correlation coefficient (r). Fig. 4A shows an example of odor-evoked responses measured simultaneously by Ca^2+^ signals, beta oscillations, high-gamma oscillations, and spikes; the correlation between Ca^2+^ and electrophysiological signals is shown in Fig. 4B. The highest correlation was between Ca^2+^ signals and beta oscillations (Fig. 4B). This finding was supported by an analysis across all animals, in which the strength of the correlation between Ca^2+^ and electrophysiological signals was in the order beta oscillations > high-gamma oscillations > spikes (Fig. 4C–F, Friedman test, beta *vs* high-gamma, *P* <0.001; high-gamma *vs* spikes, *P* <0.001; *n* = 80 from 11 mice for Ca^2+^ signals, beta , high-gamma oscillations, and spikes). These data therefore indicate that the Ca^2+^ signals recorded by fiber photometry are more closely correlated with power in the LFP beta band than with other electrophysiological signals such as spikes or high-gamma oscillations.

### Calcium Signals Show a Reliable Response to the Same Odor but Spikes Perform Best at Discriminating Different Odors

**Fig. 5:**
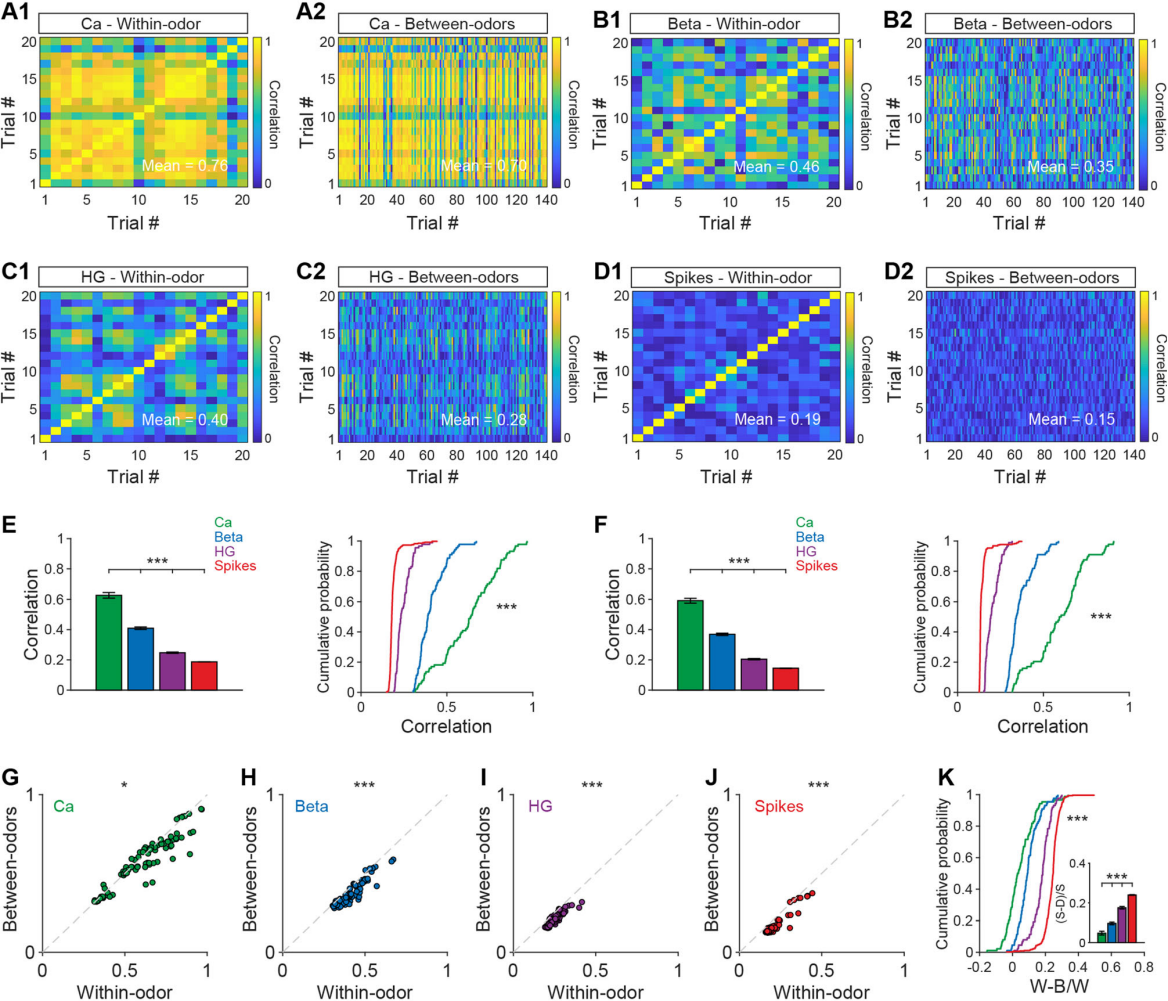
Within-odor and between-odors correlations for the Ca^2+^ signals, beta oscillations, high-gamma oscillations, and spikes. **A1**–**D1** Correlation coefficients for within-odor responses in a single mouse/cell–odor pair (20 trials) for the different types of signal. **A2**–**D2** Correlation coefficients for between-odors responses for the different types of signal. Correlations were calculated between 20 trials from one odor and 140 trials from the other 7 odors. **E, F** Statistical analysis of the correlation coefficients for within-odor (**E**) and between-odors (**F**) responses from all mouse/cell–odor pairs (left, Kruskal-Wallis test, ***P <0.001; right, two-sample K–S test, ***P <0.001; for Ca^2+^ beta and high-gamma: *n* = 88 from 11 mice; for spikes: *n* = 600 from 11 mice). **G**–**J** Comparison of the correlation coefficients for within-odor and between-odors responses from the Ca^2+^ signals (**G**), beta oscillations (**H**), high-gamma oscillations (**I**), and spikes (**J**) (Wilcoxon signed-rank test, Ca^2+^: z = 4.619, *P* = 0.010; beta: z = 8.064, *P <*0.001; high-gamma: z = 8.147, *P* <0.001; spikes: z = 16.073, *P* <0.001; for Ca^2+^, beta, and high-gamma: *n* = 88 from 11 mice; for spikes: *n* = 600 from 11 mice). The dashed diagonal lines indicate equivalent correlation coefficients in the two conditions. **K** Bar chart (Kruskal–Wallis test, ***P <0.001) and cumulative probability (two-sample K–S test, ***P <0.001) showing statistical analyses of (within-odor – between-odor)/within-odor. Ca, calcium; HG, high gamma; (W-B)/W, (within-odor – between-odor) / within-odor.

To represent odor information precisely, an individual odor should evoke a reliable neural response, and the neural response should discriminate clearly among different odors. Thus, if a signal recorded from the OB shows high reliability to the same odor on different trials and high variability to different odors, this signal is good at odor representation. To compare the reliability and variability of the Ca^2+^ and electrophysiological signals, we performed a within-odor correlation analysis (correlation between different trials with the same odor, to assess reliability, Fig. 5A1–D1) and a between-odors correlation analysis (correlation between trials for different odors, Fig. 5A2–D2). Quantitative analyses of the correlation coefficients from all mouse/cell–odor pairs showed that for within-odor correlations, the order of the correlation coefficients for the four signals was Ca^2+^ signals/beta oscillations > high-gamma oscillations > spikes (Fig. 5E, Kruskal–Wallis test, Ca^2+^ signals *vs* high-gamma, *P* <0.001; high-gamma *vs* spikes, *P <*0.001; for Ca^2+^ signals, beta, and high-gamma oscillations, *n* = 88 from 11 mice; for spikes: *n* = 600 from 11 mice). There was no statistical difference between the correlation coefficients for the Ca^2+^ signals and beta oscillations (Fig. 5E, Kruskal–Wallis test, *P >*0.05, *n* = 88 from 11 mice for both Ca^2+^ signals and beta oscillations). For between-odors correlations, the order of the correlation coefficients was Ca^2+^ signals > beta oscillations > high-gamma oscillations > spikes (Fig. 5F, Kruskal–Wallis test, Ca^2+^ signals *vs* beta, *P* = 0.03; beta *vs* high-gamma, *P <*0.001; high-gamma *vs* spikes, *P* <0.001; for Ca^2+^ signals, beta, and high-gamma oscillations: *n* = 88 from 11 mice; for spikes: *n* = 600 from 11 mice).

We also found that the within-odor correlation coefficients were higher than the between-odors coefficients for all signals recorded (Fig. 5G–J, Wilcoxon signed-rank test, Ca^2+^ signal: z = 4.619, *P* = 0.010; beta: z = 8.064, *P < *0.001; high-gamma: z = 8.147, *P* < 0.001; spikes: z = 16.073, *P* <0.001; for Ca^2+^ signals, beta and high-gamma oscillations: *n =* 88 from 11 mice; for spikes: *n* = 600 from 11 mice). This result indicates that all four types of signal responded more reliably to the same odor than to different odors; that is, they all have the ability to represent odor identity. To further assess which signal best discriminates among odors, we analyzed the difference in correlation coefficients from the within-odor and between-odor analyses. If the difference is large, the ability to discriminate odors is better. We found that spikes are the best signal for discriminating among odors, with the order spikes > high-gamma oscillations > Ca^2+^ signals/beta oscillations (Fig. 5K, Kruskal-Wallis test, spike *vs* high-gamma, *P* <0.001; high-gamma *vs* Ca^2+^ signals, *P <*0.001; for Ca^2+^ signals, beta and high-gamma oscillations, *n* = 88 from 11 mice; for spikes: *n =* 600 from 11 mice). There was no statistical difference between the Ca^2+^ signals and beta oscillations (Fig. 5K, Kruskal-Wallis test, *P* >0.05; for both Ca^2+^ signals and beta, *n* = 88 from 11 mice). These results indicate that Ca^2+^ signals have the highest reproducibility to odor stimulation in general but that spikes, which have the highest difference in correlation coefficients in the within-odor and between-odor conditions, are potentially the best signal for representing distinct odors.

**Fig. 6:**
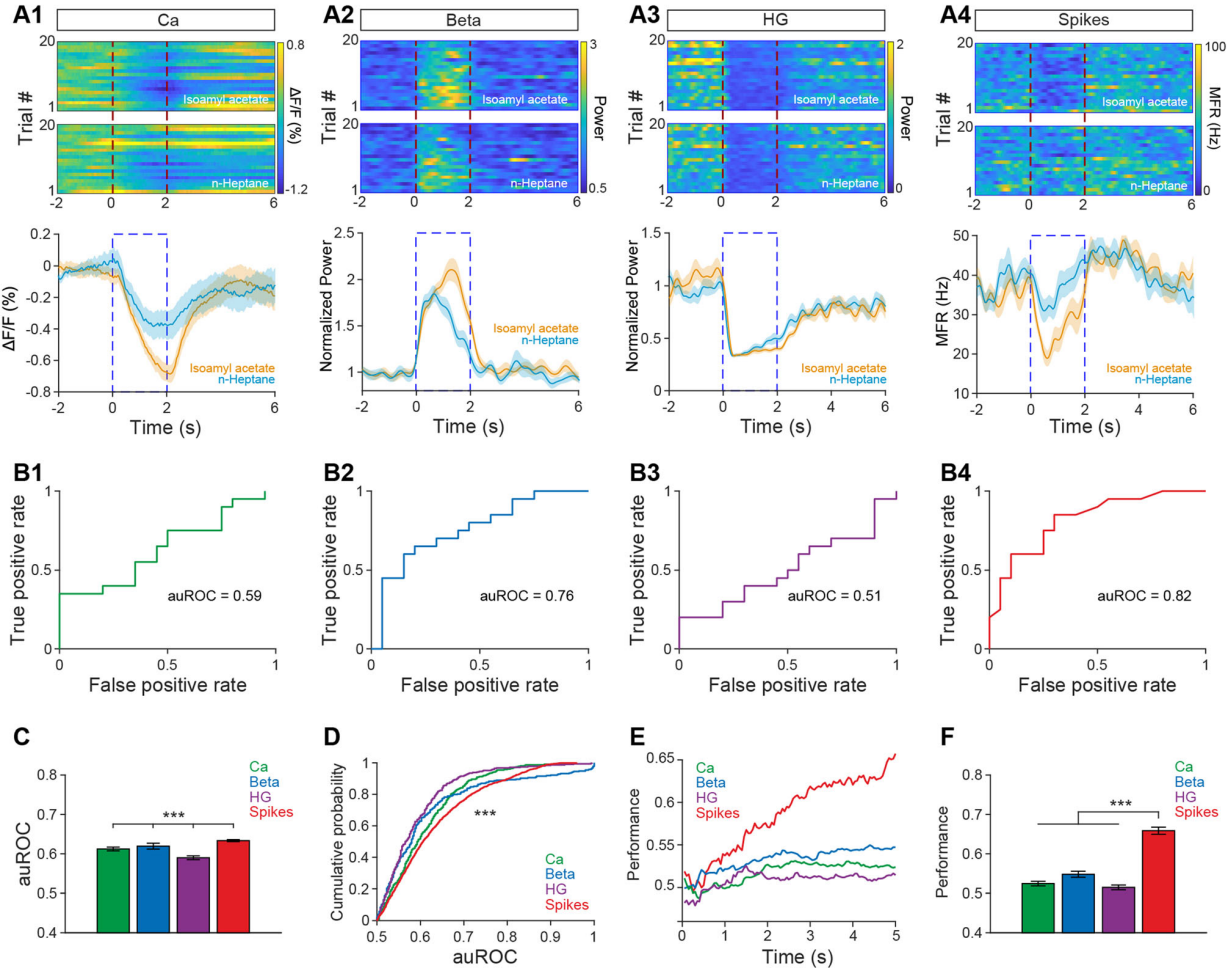
De**c**oding ability of the Ca^2+^ and electrophysiological signals. **A1**–**A4** Heat maps (upper panels, 20 trials) and trial-averaged traces (lower panels) showing the Ca^2+^ signal, power in the beta and high gamma bands, and spikes evoked by a pair of odors (isoamyl acetate *vs* n-heptane) in a representative mouse. **B** auROC analysis of the difference in ΔF/F, normalized LFP band power, and MFR induced by the two odors shown in **A**. **C, D** auROC analysis of all mouse/cell–odor pairs (**C**: Kruskal–Wallis test, ***P <0.001; **D**: two-sample K–S test, ***P <0.001; for spikes: *n =* 1652 pairs from 11 mice; for Ca^2+^, beta and high-gamma, *n* = 308 pairs from 11 mice). **E** Performance of the logistic regression classifiers on odor-pair discrimination for ΔF/F, normalized LFP band power, and MFR processed by subtracting the baseline. **F** Statistical analyses of the accuracy of all odor pairs when time = 5 s as in **E** (Kruskal–Wallis test, ***P <0.001; for spikes, sample size = 75 from 11 mice for each of the 8 odors; for Ca^2+^ signals, sample size = 220 from 11 mice for each odor; for beta and high-gamma oscillations, sample size = 200 from 11 mice for each odor). Ca, calcium; HG, high gamma

The analysis above used signal correlation, which only indirectly reflects odor discrimination. To directly analyze which signals best discriminate odors, we performed ROC analysis (Fawcett, 2006) to compare the classification of the responses evoked by odor pairs for the four types of signal. An example is shown in Fig. 6A: both odors in a pair (isoamyl acetate *versus* n-heptane) evoked robust responses, as measured by the different signals. The auROC, representing the difference in responses to the odor pair, was largest for spikes and smallest for high-gamma oscillations (Fig. 6B). ROC analysis of all animal–odor pairs or cell–odor pairs showed that the auROC values for the four types of signal had the order spikes > Ca^2+^ signals/beta oscillations > high-gamma oscillations (Fig. 6C, D, Kruskal–Wallis test, spikes *vs* Ca^2+^ signals, *P* = 0.047; Ca^2+^ signals *vs* high-gamma, *P* = 0.002; for spikes: *n* = 1652 pairs from 11 mice; for Ca^2+^ signals and high-gamma oscillations, *n* = 308 pairs from 11 mice). There was no statistical difference between Ca^2+^ signals and beta oscillations (Fig. 6C, D, Kruskal–Wallis test, *P* >0.05; for both Ca^2+^ signals and beta oscillations, *n* = 308 pairs from 11 mice). Therefore, although spikes are the best signals for classifying different odors, the Ca^2+^ signals recorded *via* fiber photometry can also be used to discriminate odors, and perform better than high-gamma LFP oscillations.

In addition, logistic regression classifiers were used to assess the performance of different types of neural responses in odor pair discrimination. Classification accuracy was calculated from an average of 28 odor pairs. The results were basically consistent with the auROC classification, which was largest for spikes and smallest for high-gamma oscillations. As the concatenated vector sets grew, the order of discrimination showed as spikes > Ca^2+^ signals/beta oscillations/high-gamma oscillations (Fig. 6E, for spikes, sample size = 75 from 11 mice for each of the 8 odors; for Ca^2+^ signals, sample size = 220 from 11 mice for each odor; for beta and high-gamma oscillations, sample size = 200 from 11mice for each odor). Statistical analyses of the accuracy with all 0–5s vectors after the onset of odor stimulation showed significant differences between the spikes and the other three types of response (Fig. 6F Kruskal–Wallis test, spikes *vs* Ca^2+^ signals, beta, and high-gamma oscillations, *P* <0.001; for spikes, sample size = 75 from 11 mice for each of the 8 odors; for Ca^2+^ signals, sample size = 220 from 11 mice for each odor; for beta and high-gamma oscillations, sample size = 200 from 11 mice for each odor). This also confirms that spikes are the best signal for discriminating among odors.

## Discussion

Fiber photometry can detect changes in fluorescent signals from a specific neuronal population surrounding the tip of the fiber and is widely used to monitor neural activity from specific brain regions in behaving animals [4–6]. Since the signal it detects is not electrical, it only indirectly reflects neural activity. Thus, understanding the correlation between Ca^2+^ signals recorded from fiber photometry and electrophysiological signals recorded by electrodes is fundamental for interpreting population Ca^2+^ signals and how they relate to neural activity. To our knowledge, this study is the first attempt to correlate population Ca^2+^ signals and electrophysiological signals in the brain. *Via* simultaneously recording with opto-tetrodes, we identified the response characteristics of Ca^2+^ signals, spikes, and beta and high-gamma LFP oscillations and assessed the ability of each signal to discriminate odors. These findings provide direct evidence of how Ca^2+^ signals recorded by fiber photometry correlate with different types of electrophysiological signal.

M/Ts are the main output neurons of the OB. They receive direct input from olfactory sensory neurons and transmit odor information to higher olfactory centers. Within the OB, M/Ts also receive inhibitory input from GABAergic interneurons located in all layers of the OB and dopaminergic interneurons in the glomerular layer [34–36]. The glomerular layer and the external plexiform layer contain two important neural circuits for mediating the M/T odor response [13, 19]. The responses of M/Ts to different odors are complex and dependent on brain state [14]. For example, although odors usually evoke an increase in single-unit M/T spikes in anesthetized animals, both excitatory and inhibitory responses are recorded in awake, behaving animals, with inhibitory responses more common than excitatory responses [25, 26, 31, 37]. The results from the present study are consistent with those from previous electrophysiological studies: whereas spikes show either inhibitory or excitatory responses depending on the odor and cell recorded, beta oscillations consistently show increases in power to odor presentation and high-gamma oscillations consistently show decreases in power [23, 24]. Moreover, the odor-evoked Ca^2+^ signals recorded *via* fiber photometry are generally consistent with our previous study, with both excitatory and inhibitory responses detected, although more inhibitory responses were found in the present study and more excitatory responses were found in the previous study [6]. This slight difference is likely due to the recording locations differing slightly in different experiments. Overall, the opto-tetrode method for simultaneously recording electrophysiological signals and Ca^2+^ signals is robust and reliable, and can be widely used in brain areas beyond the OB.

Since Ca^2+^ signals are chemical in nature, it is not surprising that they are much slower than electrical signals. However, direct comparison of the temporal pattern of Ca^2+^ signals and electrophysiological signals is important for interpreting fiber photometry data. Our data indicate that, generally, electrophysiological signals are faster than Ca^2+^ signals, since the former have shorter onset and peak latencies, and have shorter response durations (Fig. 3). Interestingly, although the differences in mean onset and peak latencies between Ca^2+^ signals and spikes were significant, the difference was small in magnitude (Fig. 3, 0.54 s *vs* 0.50 s for onset latency; 2.36 s *vs* 2.04 s for peak latency). However, this result does not indicate that the Ca^2+^ signals can be as fast as spikes; rather, this is because the temporal profiles of spike responses to odors were rather variable (Fig. 5). Variability in the M/T spike response to odors in awake animals has been reported extensively [25, 31, 38]. Spikes from a single cell reflect specific properties that vary from cell to cell but Ca^2+^ signals recorded *via* fiber photometry detect the averaged activity from a population of cells and thus do not reflect the properties of any individual cell.

How non-electrical activity correlates with electrical neural activity is a fundamental topic in neuroscience research. The correlations between vascular density, synaptic transmission, metabolism, and neurovascular coupling in optical imaging have been investigated extensively [39, 40]. Furthermore, previous studies have identified that the BOLD signal recorded during fMRI is most closely correlated with low-gamma LFP oscillations in the OB [41]. Our study reveals that the Ca^2+^ signals recorded *via* fiber photometry are most closely correlated with beta LFP oscillations. It is reasonable that the fiber photometry Ca^2+^ signal is better correlated with the LFP than with single-unit spikes since both the fiber photometry signal and the LFP signal reflect activity from a population of cells. It is interesting that the Ca^2+^ signal correlates better with beta oscillations than high-gamma oscillations. In the OB, gamma oscillations arise from interactions in the dendro-dendritic microcircuit between mitral cells and granule cells and reflect local neural network activity [24, 42, 43], whereas beta oscillations reflect activity in the wider olfactory network, including the centrifugal inputs to the OB from higher olfactory centers such as the piriform cortex [42, 44]. Our findings thus indicate that the fiber photometry Ca^2+^ signal may reflect the activity in global rather than local neural networks. Indeed, the beta oscillations and fiber photometry signals show functional similarities: in both, the odor-evoked responses are significantly modulated by learning in an odor discrimination task [6, 23, 24].

The most important task of the olfactory system is to represent odor information precisely. More and more evidence supports the hypothesis that M/Ts in the OB represent odor identity [14, 15, 20, 45]. Spikes from single M/Ts carry important information about odor identity [45, 46], although spikes that have a sniffing cycle or gamma oscillations as a frame represent odor identity more accurately [22, 47]. Thus, spikes have good features for discriminating odors. For LFP signals, both beta and gamma (especially high-gamma) oscillations are critically involved in the learning process during an odor discrimination task [24, 33, 43], and beta oscillations are thought to carry information on the chemical factors of odors [48]. However, there is no direct evidence that LFPs are a good candidate signal for representing odor identity. The present study is generally consistent with previous findings that spikes perform the best and high-gamma oscillations perform the worst in odor discrimination [6, 23]. Fiber photometry Ca^2+^ signals perform significantly worse than spikes, but can discriminate odors, performing better than gamma oscillations. This is consistent with our previous study in which Ca^2+^ signals could discriminate between a pair of odors, although this discrimination was dependent on task demands [6].

Another interesting finding in our study is that the population Ca^2+^ signal was the most reliable signal when responding to the same odor. This indicates that this signal is a good candidate for detecting odor stimulation even though its ability to discriminate odors is not strong. Since spikes perform well in discriminating odors but are not good at detecting odors, owing to their high variability, combining spike and population Ca^2+^ recordings enables both odor detection and odor discrimination to be precisely monitored. A similar method to simultaneously monitor EEG signals and fiber photometry signals was described in a recent study [49]. Thus, simultaneous recording of neural activity by electrophysiology and fiber photometry, as in the present study, is a powerful technique for studying the functions of single cells and neural circuits underlying sensory processing, cognition, and specific behaviors.

Olfactory dysfunction is closely correlated with many neurodegenerative diseases, such as Alzheimer's disease and Parkinson's disease [12]. The neural activity and odor response in the OB are significantly changed in models of these diseases, in which the function of the OB is impaired. For example, while the spontaneous spike activity is increased and odor-evoked responses in the OB are decreased in a mouse model of Alzheimer's disease [11, 50], similar findings have also been reported for LFP in the OB in a mouse model of Parkinson's disease. Thus, electrophysiological signals in the OB reflect the functional change of the OB [32]. This raises the question of whether Ca^2+^ signals recorded by fiber photometry also have the ability to reflect functional changes in the OB. This question can be addressed by recording the Ca^2+^ signals and electrophysiological signals simultaneously as in our present study in an OB lesion mouse model. Thus, direct evidence of how Ca^2+^ signals correlate with electrophysiological signals in the impaired OB is needed.

To summarize, in the present study we compared population Ca^2+^ signals with simultaneously recorded electrophysiological signals to fully assess the response characteristics, temporal correlations, and odor identity representations in the different signals. The results provide guidelines for the application of fiber photometry in other areas of neuroscience research and serve as a reminder to be cautious when interpreting Ca^2+^ signals with regard to neural activity.
